# Construction of prediction model for *KRAS* mutation status of colorectal cancer based on CT radiomics

**DOI:** 10.1007/s11604-023-01458-3

**Published:** 2023-06-14

**Authors:** Yuntai Cao, Jing Zhang, Lele Huang, Zhiyong Zhao, Guojin Zhang, Jialiang Ren, Hailong Li, Hongqian Zhang, Bin Guo, Zhan Wang, Yue Xing, Junlin Zhou

**Affiliations:** 1 Department of Radiology, Affiliated Hospital of Qinghai University, Tongren Road No. 29, Xining, 810001 People’s Republic of China; 2 Department of Radiology, Lanzhou University Second Hospital, Cuiyingmen No. 82, Chengguan District, Lanzhou, 730030 People’s Republic of China; 3 Key Laboratory of Medical Imaging of Gansu Province, Lanzhou, 730030 People’s Republic of China; 4 Gansu International Scientific and Technological Cooperation Base of Medical Imaging Artificial Intelligence, Lanzhou, 730030 People’s Republic of China; 5 The Fifth Affiliated Hospital of Zunyi Medical University, Zunyi, 519100 People’s Republic of China; 6 Department of Nuclear Medicine, Lanzhou University Second Hospital, Lanzhou, China; 7 Department of Neurosurgery, Lanzhou University Second Hospital, Lanzhou, China; 8 Sichuan Academy of Medical Sciences & Sichuan Provincial People’s Hospital, Chengdu, China; 9 Department of Pharmaceuticals Diagnosis, GE Healthcare, Beijing, China; 10 Affiliated Hospital of Qinghai University, Xining, China; 11 Xinxiang Medical University, Henan, China

**Keywords:** *KRAS* mutation, Colorectal cancer, Radiomics, CT, Triphasic enhanced phase

## Abstract

**Background:**

In this study, we used computed tomography (CT)-based radiomics signatures to predict the mutation status of *KRAS* in patients with colorectal cancer (CRC) and to identify the phase of radiomics signature with the most robust and high performance from triphasic enhanced CT.

**Methods:**

This study involved 447 patients who underwent *KRAS* mutation testing and preoperative triphasic enhanced CT. They were categorized into training (*n* = 313) and validation cohorts (*n* = 134) in a 7:3 ratio. Radiomics features were extracted using triphasic enhanced CT imaging. The Boruta algorithm was used to retain the features closely associated with *KRAS* mutations. The Random Forest (RF) algorithm was used to develop radiomics, clinical, and combined clinical–radiomics models for *KRAS* mutations. The receiver operating characteristic curve, calibration curve, and decision curve were used to evaluate the predictive performance and clinical usefulness of each model.

**Results:**

Age, CEA level, and clinical T stage were independent predictors of *KRAS* mutation status. After rigorous feature screening, four arterial phase (AP), three venous phase (VP), and seven delayed phase (DP) radiomics features were retained as the final signatures for predicting *KRAS* mutations. The DP models showed superior predictive performance compared to AP or VP models. The clinical–radiomics fusion model showed excellent performance, with an AUC, sensitivity, and specificity of 0.772, 0.792, and 0.646 in the training cohort, and 0.755, 0.724, and 0.684 in the validation cohort, respectively. The decision curve showed that the clinical–radiomics fusion model had more clinical practicality than the single clinical or radiomics model in predicting *KRAS* mutation status.

**Conclusion:**

The clinical–radiomics fusion model, which combines the clinical and DP radiomics model, has the best predictive performance for predicting the mutation status of *KRAS* in CRC, and the constructed model has been effectively verified by an internal validation cohort.

**Supplementary Information:**

The online version contains supplementary material available at 10.1007/s11604-023-01458-3.

## Introduction

Colorectal cancer (CRC) is the second leading cause of cancer-related deaths worldwide and causes almost 881,000 deaths every year [1]. The incidence of colorectal cancer is approximately threefold higher in developed countries than in developing countries. However, as the developing countries become richer, increasing trends are likely to be seen [2]. The Kirsten rat sarcoma (*KRAS*) viral oncogene homolog is a G protein, which occurs in 40–50% cases of CRCs. Following a mutation in the *KRAS* gene, the mutant protein activates the downstream mitogen-activated protein kinase (MAPK) pathway, subsequently leading to uncontrolled cell proliferation and malignancy [3]. The National Comprehensive Cancer Network (NCCN) clinical practice guidelines have explicitly indicated that patients with CRC and *KRAS* mutations are resistant to anti-EGFR monoclonal antibody therapy [4]. Therefore, *KRAS* mutation testing is crucial for individualized and effective treatment of CRC.

Generally, pathologic specimens obtained via invasive procedures, such as colonoscopy and surgery, are usually required for the identification of *KRAS* mutation status. However, the presence of extensive heterogeneity in CRC archival samples represents a major limitation of the histological approach [5]. Additionally, tissue specimens for genetic testing cannot be obtained for selected patients with metastatic CRC because they cannot undergo surgical treatment [6]. Furthermore, biopsy testing might not be an effective approach to determine the mutational status of *KRAS* due to poor DNA quality [7]. Therefore, it is necessary to develop a non-invasive and easy-to-use method to identify *KRAS* mutation status.

Several studies have demonstrated the use of medical imaging technology, such as fluorine-18 fluorodeoxyglucose (18F-FDG) positron emission tomography (PET) and magnetic resonance imaging, in the prediction of *KRAS* status [8, 9]. However, these studies involved small sample sizes and lacked validation. Radiomics provide a variety of parameters for quantitative analysis, which have been widely used in cancer diagnosis, classification, and prediction [10]. A previous study demonstrated a significant correlation between a CT-based radiomics signature and *KRAS/NRAS/BRAF* mutations in patients with CRC [11]. However, this study involved a small sample size and was only performed in the venous phase (VP). Moreover, the superiority of VP compared to the arterial phase (AP) or delay phase (DP) in the prediction of *KRAS* mutation status in patients with CRC remains to be confirmed. The aim of this study was to investigate whether a CT-based radiomics signature could identify *KRAS* mutation status in patients with CRC and whether the VP is superior to arterial and delay phases in the prediction of *KRAS* mutation status in patients with CRC.

## Materials and methods

### Patients

**Fig. 1:**
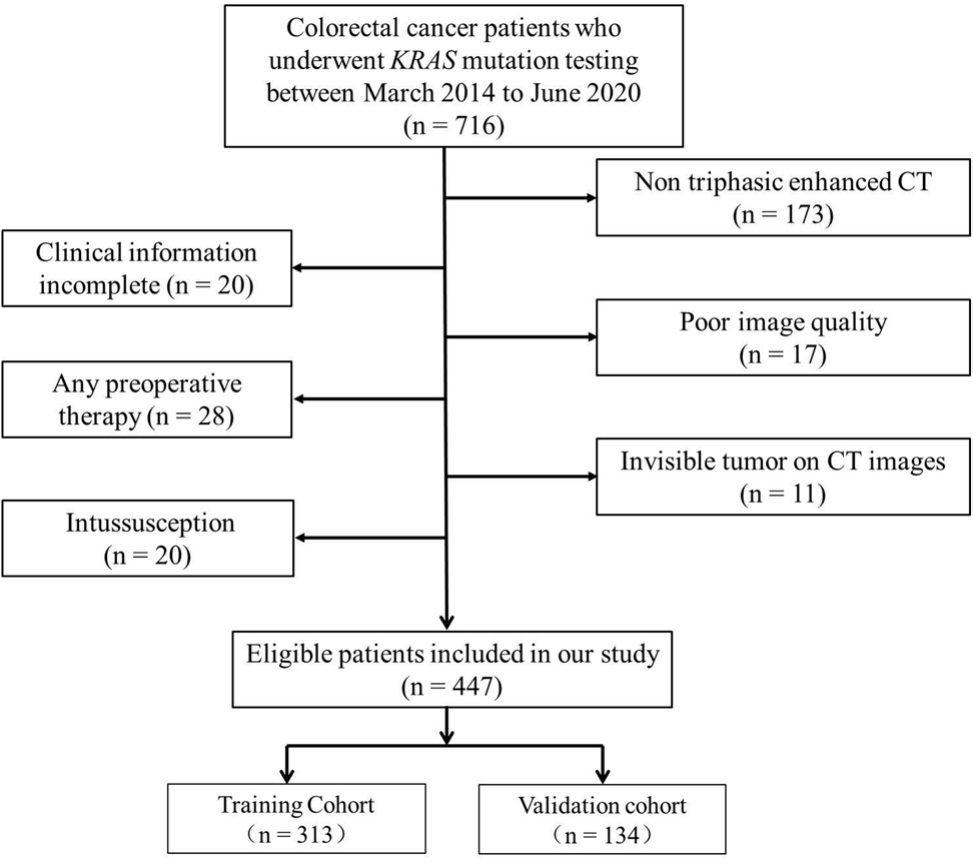
Flow diagram of the recruitment pathway

Ethical approval has been obtained by this retrospective study, and the informed consent requirement was waived. For the primary cohort of this study, we analyzed the institutional database in Lanzhou University Second Hospital between March 2014 and June 2020 to identify eligible patients with confirmed cases of CRC who underwent curative resection. A total of 447 patients met the inclusion criteria in our study, which were set based on the following factors: (1) pathologically identified cases of primary CRC adenocarcinoma; (2) patients who underwent *KRAS* mutation status testing prior to the treatment; and (3) pre-treatment abdominal triphasic enhanced CT with a reconstruction slice thickness of 1.25 mm. The exclusion criteria were set based on the following factors: (1) abdominal triphasic enhanced CT was not performed before surgery or the interval between abdominal triphasic enhanced CT and surgery was > 2 weeks; (2) patients with CRC who have received any anticancer treatment prior to the collection of pathological tissue samples; (3) insufficient CT quality for qualitative and quantitative analyses; (4) incomplete clinical information; and (5) occurrence of intussusception in the area where the tumor was located. Figure 1 shows a flow diagram of the recruitment pathway. Patients were categorized into training and validation cohorts in the ratio 7:3.

### Clinicopathological characteristics and semantic features

Baseline clinicopathological characteristics data that were collected from medical records included age, sex, tumor location, *KRAS* mutation status, CEA level (threshold value ≥ 5 ng/mL, < 5 ng/mL), CA125 level (threshold value ≥ 35 U/mL, < 35 U/mL), and CA19-9 level (threshold value ≥ 37 U/mL, < 37 U/mL). Two experienced gastrointestinal radiologists (Y T C and J Z) analyzed the CT images (including tumor location, maximum diameter, clinical tumor (cT) stage, and clinical node (cN) stage). Both radiologists were blinded to the patient’s clinicopathological data. To minimize bias, qualitative data were obtained three times and the average was calculated; qualitative data were independently evaluated and resolved through consultation when opinions were inconsistent. The maximum tumor thickness was defined as the maximum diameter perpendicular to the long axis of the cross-sectional image. The cT and cN stages were identified according to the eighth edition of the American Joint Committee on Cancer Staging System [12].

### *KRAS* mutation status evaluation

Formalin-fixed tumor tissue samples were obtained following CRC surgeries and confirmed that the specimens used to extract DNA are clearly infiltrated by the tumor. *KRAS* mutation status (exons 2, 3, and 4) was detected via polymerase chain reaction (PCR).

### CT image acquisition and segmentation

Abdominal triphasic enhanced CT scans were performed on a Discovery CT 750 HD scanner (GE Healthcare, Waukesha, WI) and an iCT 256 scanner (Philips, Amsterdam, Netherlands). The scanning parameters are listed in Supplementary Table S1. Enhanced CT scanning was performed using a high-pressure dual-cylinder syringe to inject intravenous iohexol (1 mL/kg) through the median cubital vein with an injection rate of 3.5–4.5 mL/second. Following the injection of the contrast medium, AP, VP, and DP were scanned at 25–30 s, 60–70 s, and 120–150 s, respectively.

The original images of AP, VP, and DP were stored in the corresponding folders in DICOM format. Two gastrointestinal radiologists (reader 1: Y T C and reader 2: J Z) performed three-dimensional (3D) radiomics segmentation on AP, VP, and DP images using ITK-SNAP software (version 3.6.0; www.itksnap.org). Reader 1 segmented 247 cases and reader 2 segmented the other 200 cases.

**Fig. 2:**
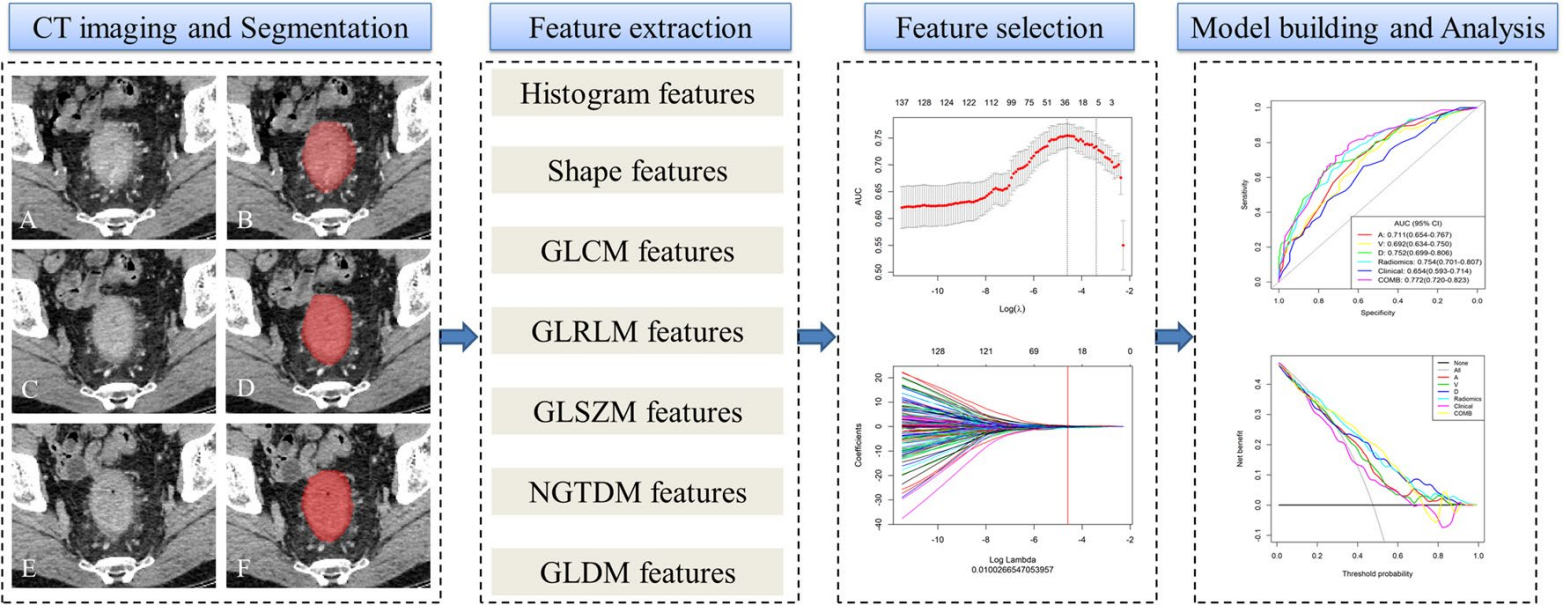
Workflow of *KRAS* prediction building and analysis. The tumors were segmented on arterial phase (**A**, **B**), delayed phase (**C**, **D**) and venous phase (**E**, **F**) CT images to form volumes of interest (VOIs). One thousand and thirty-seven quantitative radiomics features were extracted from each patient. The least absolute shrinkage and selection operator (LASSO) was used to select the features. Multivariate logistic regression was used to build radiomics, clinical, and clinicoradiomics combined models for *KRAS* prediction. Finally, the radiomics signature and clinical factors were incorporated into a nomogram for individual evaluation. Receiver operating characteristic curves were used to evaluate the clinical usefulness of the nomogram

For 3D radiomics segmentation, the ROI was manually delineated on each slice of the tumor. Air and feces in the intestinal tract and pericolonic fat were carefully excluded from the contours (Fig. 2). Finally, each patient generated three ROIs (AP ROI, VP ROI, and DP ROI). To evaluate the inter-observer reproducibility and robustness of the feature extraction, reader 1 and reader 2 randomly selected 30 patients and performed manual segmentation. We estimated the reproducibility of the feature extraction using intra-/inter-class correlation coefficients (ICCs); ICC values greater than 0.80 indicate good reproducibility [13]. Additionally, 30 patients were randomly selected from each CT scanner to build the CT scanners set for calculating the ICCs.

### Feature extraction

Radiomics features were extracted and selected using PyRadiomics software [14]. Seven classes of radiomics features (first-order histogram, 3D morphologic, gray level co-existence matrix (GLCM), gray level range-matrix (GLRM), gray level size zone matrix (GLSZM), neighboring grey tone difference matrix (NGTDM), and grey level dependence matrix (GLDM) features) were extracted from original and filtered images (wavelet and Laplacian of Gaussian). Finally, 1037 3D radiomics features were extracted from each phase of the triphasic enhanced CT. The specific definitions and descriptions of the features are demonstrated in the Supplementary Materials.

### Feature selection and radiomics prediction model building

After radiomics features extraction, all missing data in the training cohort were replaced by the median value and z-score normalization was performed on each feature; the same preprocessing procedure was applied to the validation cohort. After preprocessing of the features, the most important features were selected to predict *KRAS* mutations using a three-step procedure. First, univariate analysis was performed for feature selection to retain the feature with *P* < 0.05 to enter the following process. Second, the Boruta method [15] was used to retain the features that closely associated with *KRAS* mutations. Finally, multivariable stepwise regression further eliminated irrelevant features and retained the most informative features. A ten times fivefold cross-validation was applied to avoid overfitting and identify the model with the best performance.

Three radiomics models (AP, VP, and DP model) were established based on the above radiomics signatures in triphasic enhanced CT phase images. For example, the VP model was built based on VP features in 3D segmentation patterns (three features). Further, the 3D-combined model was built based on AP, VP, and DP fusion features in 3D segmentation patterns (11 features).

### Clinical and combined model construction

For clinical and imaging characteristics, the Chi-squared test or Fisher’s exact test were used to compare the differences in sex, tumor location, CEA, CA125, CA19-9, cT stage, and cN stage, whereas the Student’s *t* test or Mann–Whitney *U* test was used to compare the differences in age and maximum diameter between mutated *KRAS* and wild-type *KRAS* groups in the training cohorts. We performed multivariable analyses to identify the most important features. A clinical model was established based on the inclusion of selected features.

A clinical–radiomics fusion model was developed based on correlated clinical risk factors, strongly correlated imaging characteristics, and radiomics features to verify whether the combination of radiomics signatures and clinical factors could improve performance in the prediction of *KRAS* mutations. Two steps were followed to build the fusion model in this study. First, AP, VP, and DP models were compared to determine the enhancement phase with the best *KRAS* mutation prediction performance. Second, the Random Forest (RF) algorithm was used to combine clinical factors, imaging characteristics, and the radiomics features of the best predictive performance phase to construct a clinical–radiomics fusion model in the training cohort, and the discrimination ability of the fusion model was evaluated based on the AUC value in the validation cohort.

### Statistical analysis

All statistical analyses were conducted using the R statistical software package (version 3.6.3; http://www.Rproject.org). The Student’s *t* test, Mann–Whitney *U* test, and Chi-squared test or Fisher’s exact test were used to compare continuous and categorical variables, as appropriate. A two-sided *P* value < 0.05 was considered statistically significant. The ICCs were used to calculate the consistency of measurements between the two radiologists and the different CT scanners. ROC analysis was used to evaluate the predictive accuracy of the different models. The AUC value, 95% confidence interval (CI), accuracy, sensitivity, specificity, positive predictive value (PPV), and negative predictive value (NPV) were also calculated. A calibration curve was constructed to assess the goodness-of-fit of the models. To verify the clinical usefulness of the models, we quantified the net benefit at different threshold probabilities in the dataset using DCA curves.

## Results

### Clinical characteristics

This study involved a total of 447 patients with CRC in the final analysis, including 263 men (58.8%) and 184 women (41.2%), with an average age of 58.93 ± 12.85 years. Among the 447 patients, 207 had mutated *KRAS* and 240 had wild-type *KRAS.* We used stratified sampling to categorize the study cohort into training (*n* = 313) and validation (*n* = 134) cohorts in a 7:3 ratio. The training and validation cohorts were used for model building and internal validation, respectively. Patient and tumor characteristics in the training cohort are listed in Table 1.

**Table 1:** Demographic and clinical characteristics of and CT findings for CRC (mean ± SD or no. (%)

Characteristics^a^	Training cohort (*n* = 313)		
	*KRAS* WT (*n* = 164)	*KRAS* MT (*n* = 149)	*P* value^b^
Age (years)	57.58 ± 12.81	60.50 ± 12.75	0.023
Gender			
Female	63 (38.4%)	66 (44.3%)	0.291
Male	101 (61.6%)	83 (55.7%)	
Tumor location			
Left	115 (70.1%)	100 (67.1%)	0.567
Right	49 (29.9%)	49 (32.9%)	
CEA level			
Normal	101 (61.6%)	72 (48.3%)	0.018
Abnormal	63 (38.4%)	77 (51.7%)	
CA125 level			
Normal	150 (91.5%)	135 (90.6%)	0.790
Abnormal	14 (8.5%)	14 (9.4%)	
CA19-9 level			
Normal	120 (73.2%)	90 (60.4%)	0.016
Abnormal	44 (26.8%)	59 (39.6%)	
cT stage			
T1	2 (1.2%)	9 (6.0%)	0.023
T2	21 (12.8%)	29 (19.5%)	
T3	107 (65.2%)	90 (60.4%)	
T4	34 (20.7%)	21 (14.1%)	
cN stage			
N0	100 (61.0%)	81 (54.4%)	0.281
N1	37 (22.6%)	33 (22.1%)	
N2	27 (16.5%)	35 (23.5%)	
Maximum diameter (mm)	22.42 ± 9.95	21.85 ± 8.88	0.825

*WT* wild type, *MT* mutated type

^a^Continuous variables were expressed as mean ± standard deviation; classification variables were represented by no. (%)

^b^Student’s *t* test or Mann–Whitney *U* test was used to compare continuous variables; Chi-squared test or Fisher’s exact test was used to compare categorical variables

### Predictive performance of the clinical model

In the training cohort, the clinical characteristics age, CEA, CA19-9, and cT stage were found to be significantly different statistically (*P* < 0.05), and the other characteristics not significantly different (*P* > 0.05) between mutated *KRAS* and wild-type *KRAS* groups (Table 1). After multivariate analyses, clinical characteristics including age, CEA, and cT stage were selected as independent predictors of *KRAS* mutation and enrolled into clinical model. The clinical model showed lower performance in predicting *KRAS* mutation both in the training cohort and the validation cohort, with the AUC being 0.654 (95% CI 0.593–0.714) in the training cohort and 0.575 (95% CI 0.478–0.672) in the validation cohort (Table 2). The accuracy, sensitivity, and specificity were 0.617, 0.664, and 0.573 (training cohort) and 0.552, 0.552, and 0.553 (validation cohort), respectively.

**Table 2:** Predictive performance of different models in training and validation cohorts

Feature_num	Methods	Training cohort						Validation cohort					
		AUC	Accuracy	Sensitivity	Specificity	PPV	NPV	AUC	Accuracy	Sensitivity	Specificity	PPV	NPV
4	A	0.711	0.649	0.758	0.549	0.604	0.714	0.723	0.679	0.793	0.592	0.597	0.789
3	V	0.692	0.639	0.597	0.677	0.627	0.649	0.673	0.657	0.621	0.684	0.600	0.703
7	D	0.752	0.709	0.638	0.774	0.720	0.702	0.746	0.687	0.569	0.776	0.660	0.702
11	Radiomics	0.754	0.700	0.738	0.665	0.667	0.736	0.775	0.701	0.707	0.697	0.641	0.757
3	Clinical	0.654	0.617	0.664	0.573	0.586	0.653	0.575	0.552	0.552	0.553	0.485	0.618
4	Clinical–radiomics	0.772	0.716	0.792	0.646	0.670	0.774	0.755	0.701	0.724	0.684	0.636	0.765

*Radiomics* fusion of radiomics features of arterial phase, venous phase, and delayed phase; *clinical* fusion of clinical and imaging characteristics; *clinical–radiomics* fusion of clinical radiological features and radiomics features, *AP* radiomics model of arterial phase, *DP* radiomics model of delayed phase, *VP* radiomics model of venous phase, *AUC* area under the curve, *NPV* negative predictive value, *PPV* positive predictive value

### Radiomics signature building and discrimination performance assessment

**Fig. 3:**
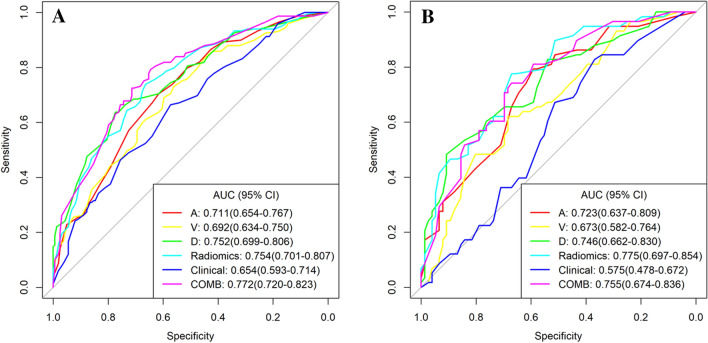
ROC curves of the different models in training (**A**) and validation cohorts (**B**). *AUC* area under the curve, *A* radiomics model of arterial phase, *D* radiomics model of delayed phase, *V* radiomics model of venous phase, *Radiomics* radiomics model of fusion of arterial phase, delayed phase and venous phase features, *COMB* fusion of clinical risk factors and radiomics features of delayed phase

The ICCs were calculated to evaluate the agreement of features extracted by two radiologists and different CT scanners; all values > 0.80 indicate good agreement. A total of 1037 3D radiomics features were extracted from each patient's AP, VP, and DP images. Finally, four, three, and seven radiomics features were selected as the final signatures. The feature names and distributions are listed in Table 3. Following stepwise regression analysis, three features were removed after combining the AP, VP, and DP radiomics features. Four models were built based on the above radiomics signatures for preoperatively predicting *KRAS* mutations. The AUC, accuracy, sensitivity, specificity, PPV, and NPV are listed in Table 2. The DP model had the most optimal predictive performance compared to the AP or VP model in both the training and validation cohorts (Fig. 3A, B, Table 2). In the training cohort, the predictive AUC of *KRAS* mutations in AP, VP, and DP models were 0.711, 0.692, and 0.752, respectively. In the validation cohort, the AUC of the three models were 0.723, 0.673, and 0.746, respectively. The radiomics model combined with the triphasic enhanced CT phases showed moderate *KRAS* mutation prediction performance, with an AUC, accuracy, sensitivity, specificity, PPV, and NPV of 0.754, 0.700, 0.738, 0.665, 0.667, and 0.736 in the training cohort, respectively, whereas the AUC, accuracy, sensitivity, specificity, PPV, and NPV in the validation cohort were 0.775, 0.701, 0.707, 0.697, 0.641, and 0.757, respectively (Fig. 3A, B, Table 2).

**Table 3:** The final signatures selected from 3D radiomics features

Arterial phase (4)	Venous phase (3)	Delayed phase (7)	Radiomics (11)
A_original_shape_Elongation	V_original_shape_Maximum2DDiameterSlice	D_original_shape_Elongation	A_original_shape_Elongation
A_wavelet.HLL_firstorder_Skewness	V_original_shape_Sphericity	D_original_shape_Sphericity	A_wavelet.HHL_firstorder_Skewness
A_wavelet.HHH_glszm_GrayLevelNonUniformityNormalized	V_wavelet.HLL_ firstorder_Median	D_wavelet.HLL_glcm_Idn	A_wavelet.HHH_glszm_GrayLevelNonUniformityNormalized
A_wavelet.LLL_glcm_MCC		D_wavelet.LLL_glcm_Idn	A_wavelet.LLL_glcm_MCC
		D_original_shape_Maximum3DDiameter	V_original_shape_Maximum2DDiameterSlice
		D_original_shape_SurfaceArea	D_original_shape_Elongation
		D_wavelet.HLL_gldm_SmallDendenceLowGrayLevelEmphasis	D_original_shape_Maximum3DDiameter
			D_original_shape_Sphericity
			D_wavelet.HLL_glcm_Idn
			D_wavelet.HLL_gldm_SmallDendenceLowGrayLevelEmphasis
			D_wavelet.LLL_glcm_Idn

### Predictive performance of the combined model

**Fig. 4:**
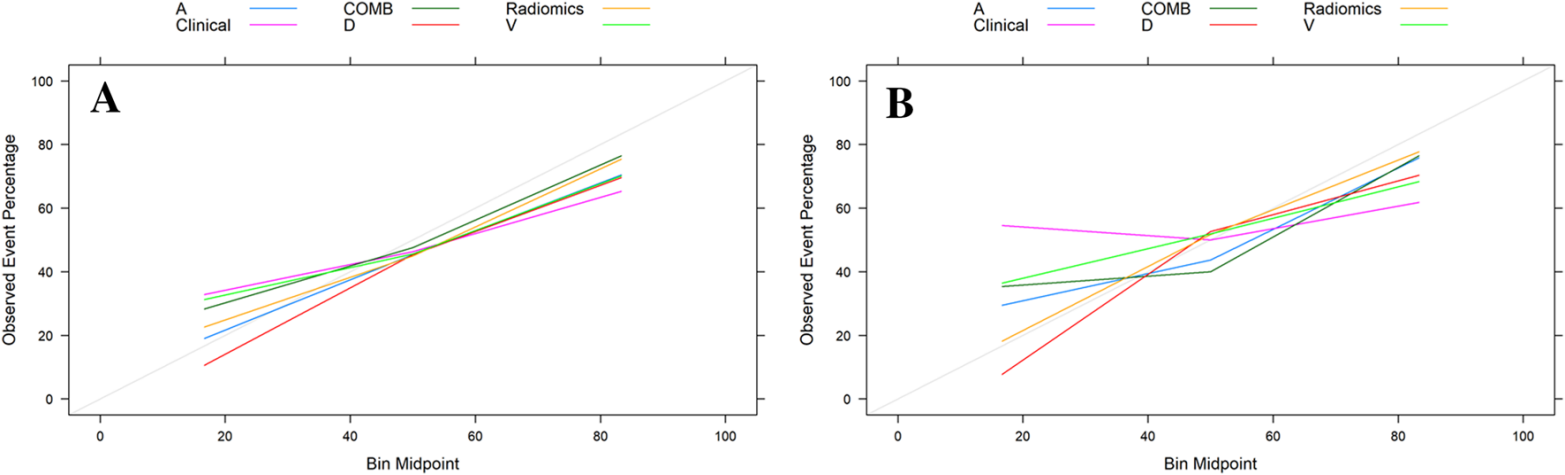
Calibration curves of the different models in training (**A**) and validation cohorts (**B**). *A* radiomics model of arterial phase, *D* radiomics model of delayed phase, *V* radiomics model of venous phase, *Radiomics* radiomics model of fusion of arterial phase, delayed phase and venous phase features, *COMB* fusion of clinical risk factors and radiomics features of delayed phase

As shown in Table 2 and Fig. 3, we developed a clinical–radiomics model incorporating three clinical factors (age, CEA, and cT stage) and seven DP radiomics signatures. The clinical–radiomics model showed excellent predictive ability for *KRAS* mutations. The clinical–radiomics fusion model showed superior predictive performance for *KRAS* mutations compared to either the clinical model or the radiomics model alone; the AUC values of the clinical–radiomics model were 0.772 (95% CI 0.720–0.823) in the training cohort and 0.755 (95% CI 0.674–0.836) in the validation cohort. The calibration curve of each model showed favorable agreement between prediction and observation in predicting the risk of *KRAS* mutations (Fig. 4A, B).

**Fig. 5:**
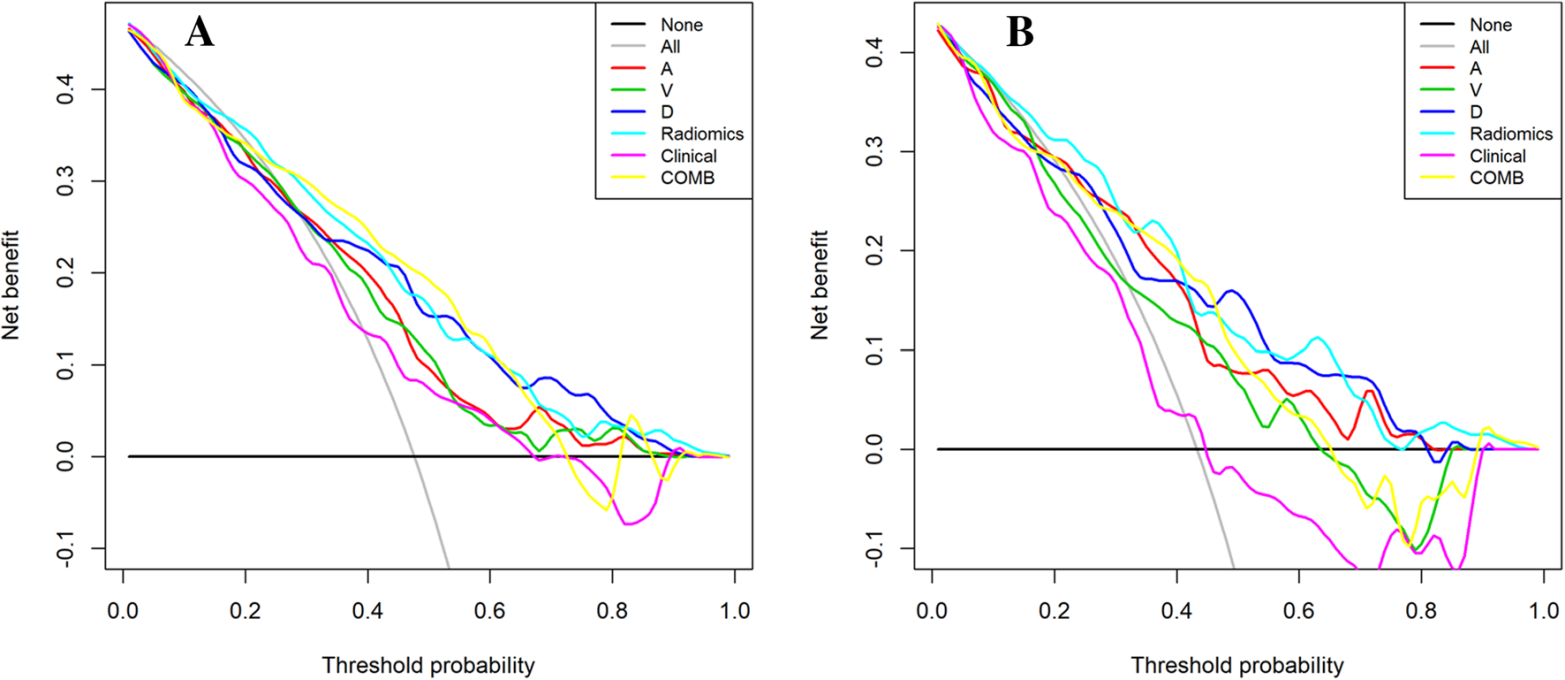
Decision curve analysis of different models in training (**A**) and validation cohorts (**B**). *A* radiomics model of arterial phase, *D* radiomics model of delayed phase, *V* radiomics model of venous phase, *Radiomics* radiomics model of fusion of arterial phase, delayed phase and venous phase features, *COMB* fusion of clinical risk factors and radiomics features of delayed phase

The DCA curves for the clinical model, radiomics model, and clinical–radiomics model are presented in Fig. 5A, B. The clinical–radiomics model achieved more clinical utility in predicting *KRAS* mutations than the clinical model and other radiomics models. The DCA curves of the clinical–radiomics model demonstrated that when the threshold probability of a patient or doctor ranged between 20 and 65%, the use of the clinical–radiomics nomogram adds greater benefit for *KRAS* mutation prediction than the treat-all-patients scheme or the treat-none scheme in the training and validation cohorts.

## Discussion

In this study, clinical, radiomics, and clinical–radiomics models were developed for the preoperative prediction of *KRAS* mutations. We verified our hypothesis that the DP model had a higher predictive performance than the AP or VP models. Additionally, the clinical–radiomics model showed a higher predictive performance than the clinical or radiomics models alone. The calibration and decision curves of the clinical–radiomics model showed excellent model stability and actual benefit.

KRAS mutations can lead to continuous activation of the EGF/RAS/RAF/ERK signaling pathway without the regulation of EGFR, gradually leading to increased cell proliferation and decreased apoptosis [16–18]. Colorectal cancer with *KRAS* mutations is a negative marker for anti-EGFR targeted drugs [19]. Numerous studies have used 18F-FDG PET/CT to investigate the association between *KRAS* mutations and 18F-FDG uptake and demonstrated that cells with *KRAS* mutations had a higher 18F-FDG uptake than those with wild-type *KRAS* [20, 21]. However, there was no correlation observed between them according to a study by Riklis et al. [22]. The major clinical use of PET/CT in CRC is to detect potentially curable metastases. Yang et al. [11] proposed a CT-based radiomics model to identify *KRAS/NRAS/BRAF* mutations in CRC and found a relatively high predictive performance. However, this study defined the positive group based on mutations in any of *KRAS/NARS/BRAF*, which would complicate the clinical application.

In the present study, the clinical model constructed by clinicopathological factors has the ability to identify *KRAS* gene mutations. However, the diagnostic value of the clinical model was quite low. When the clinicopathological factors and DP radiomics features were combined as a clinical–radiomics model, the predictive AUC value of the clinical–radiomics model reached 0.772 in the training cohort and 0.755 in the validation cohort. Thus, the radiomics features can provide additional benefits in predicting *KRAS* mutations.

During the image preprocessing stage, the LoG and wavelet filters were applied to process the original image. The LoG filter can smoothen the image and improve the efficiency of capturing phenotypic features related to tumor heterogeneity [23]. The wavelet filter could disassemble the frequency signal of the image to extract edges and substantial features of the tumor more effectively. This study finally screened out 25 radiomics features as the radiomics signatures of the AP, VP, DP, and triphasic enhanced combined phase. The features with wavelet filtering accounted for 52% (13/25) of the total features. This shows that the wavelet filter is important for extracting features related to *KRAS* mutation status, whereas the features with LoG filters are not used as radiomics signatures for predicting *KRAS* mutations, indicating that the features extracted from the LoG filter images were weakly correlated with *KRAS* mutations.

Using multivariable regression analysis combined with the radiomics signatures of the triphasic enhanced phases, 11 radiomics features were retained as key features for identifying *KRAS* mutation status (Table 3), including 5 texture features: A_wavelet.HHH_glszm_GrayLevelNonUniformityNormalized, A_wavelet.LLL_glcm_MCC, D_wavelet.HLL_glcm_Idn, D_wavelet.HLL_gldm_SmallDendenceLowGrayLevelEmphasis, and D_wavelet.LLL_glcm_Idn. Texture features are microscopic tumor descriptions, which reflects the interaction between adjacent pixels as well as tumor heterogeneity [24]. These features are not easily identified by the human visual system and cannot be interpreted as having a clear meaning. Previous studies have shown that texture features may be associated with the tumor microenvironment reflecting tumor heterogeneity and the presence of hypoxia or angiogenesis [25–27]. A previous study found that *KRAS* mutations were associated with higher texture characteristic values (Gskewness and SDs), indicating that mutated *KRAS* had more tumor heterogeneity than wild-type *KRAS* [28]. The radiomics score values of texture features (A_wavelet. LLL_glcm_MCC and D_wavelet. HLL_gldm_SmallDendenceLowGrayLevelEmphasis) in the *KRAS* mutation group were higher than that in the wild-type group, which suggests more tumor heterogeneity in the tumor tissue in the ROI range. The results of this study in combination with that of other studies shows that texture features can be used as non-invasive imaging markers for predicting *KRAS* mutations status.

In addition, five morphological features (A_original_shape_Elongation, V_original_shape_Maximum2DDiameterSlice, D_original_shape_Elongation, D_original_shape_Maximum3DDiameter, and D_original_shape_Sphericity) were closely correlated with *KRAS* mutations, suggesting that the morphological characteristics of mutant *KRAS* and wild-type *KRAS* tumors were significantly different, which is consistent with previous literatures. A previous study found that morphological features (elongation and flatness) were closely associated with *KRAS* mutations in rectal cancer [23]. Another study explored the correlation between *KRAS* mutations in rectal cancer and tumor morphology in magnetic resonance images, and found that the average axial/longitudinal ratio of *KRAS* mutations in rectal cancer was greater than that of *KRAS* wild-type tumors (0.46 ± 0.29 vs. 0.36 ± 0.20, *P* = 0.009) [9].

Among the triphasic enhanced phase models of *KRAS* mutation prediction in the training cohort, the DP model showed the highest performance, with an AUC value of 0.752, followed by 0.711 in the AP model and 0.692 in the VP model. To our knowledge, this is the first time that triphasic enhanced CT radiomics has been used in *KRAS* mutation prediction. Although the VP is the most commonly used phase in gastrointestinal radiomics research, contrary to the results observed in this study, the enhancement phase with the best predictive performance was the DP rather than the VP phase. The high predictive performance of the DP model might be due to the possibility of high content and uniform distribution of the contrast agent in the DP lesions or because the ROI range of tumors in the DP images is larger than that in the AP and VP images [29].

In terms of clinical characteristics, age, CEA, and CA19-9 were independent predictors for *KRAS* mutations. In this study, patients with *KRAS* mutations were significantly older than those with *KRAS* wild-type *P* < 0.05, which is consistent with the findings of a previous study [30]. CEA and CA19-9 were significantly higher for the mutated *KRAS* group than for the wild-type *KRAS* group in our study, which is in line with findings from previous studies [31, 32]. Both *KRAS* mutations and elevated serum levels of CEA and CA19-9 are associated with more aggressive biological behavior in patients with CRC [33–35]. A correlation between *KRAS* mutations and higher CEA and CA19-9 levels suggests that genetic alterations may have independent influences on CRC development, thus resulting in increased tumor biomarkers [36].

Triphasic enhanced CT is often conducted in CT examination of gastrointestinal tumors. The AP is used for tumor detection, the VP to differentiate the tumor from adjacent organs, and the DP to determine the depth of tumor invasion [37]. As for radiation dose, the average DLP value of triphasic enhanced scans was 1917.52 ± 152.31 mGy cm, which is slightly higher than the diagnostic reference level for adults (1490 mGy cm) published by China's National Health Industry standard (WS/T 637-2018) [38]. Application of new techniques such as multi-model iterative reconstruction technology could effectively reduce the radiation dose in clinical practice [39].

Our study should be interpreted after considering several limitations. First, 269 patients were excluded because they did not meet the inclusion and exclusion criteria, which inevitably produced a selection bias. Second, our study only included a single team with an internal validation cohort. The reproducibility should be addressed in future multi-center studies. Third, due to the irregular shape of some tumors, the ROI delineation process is difficult and time consuming. In future studies, it will be necessary to develop an automated or semi-automated tool to achieve effective and automatic tumor segmentation. Finally, we used different imaging instruments and acquisition parameters to complete the CT scanning. The influence of different instruments and different parameters on radiomics features is obvious. Therefore, it is important to standardize scanning protocols in different instruments and different institutions.

## Conclusion

In conclusion, triphasic enhanced CT radiomics models were constructed to predict *KRAS* mutation status in colorectal cancer, and the results showed that the AP, VP, and DP models could better predict *KRAS* mutation status in the training and validation cohorts. The DP model showed a higher predictive performance compared to the AP or VP models. Additionally, the clinical–radiomics model, which incorporates both clinical risk factors and radiomics features of DP images, showed good performance in predicting *KRAS* mutations. The clinical–radiomics fusion model can be used as a potential imaging marker for preoperative detection of *KRAS* mutation status and guide the selection of molecular targeted drug therapy for CRC.

### Supplementary Information

Below is the link to the electronic supplementary material.

Supplementary file1 (DOCX 19 KB)

## Abbreviations

AP: Arterial phase
CRC: Colorectal cancer
DP: Delayed phase
*KRAS*: Kirsten rat sarcoma
VP: Venous phase
3D: Three-dimensional
