# Supplementary material for: Construction of prediction model for KRAS mutation status of colorectal cancer based on CT radiomics
Source: Jpn J Radiol. 2023 Jun 14;41(11):1236–46. doi: 10.1007/s11604-023-01458-3 (PMC10613595; doi:10.1007/s11604-023-01458-3)
Supplement: Supplementary file 1 — Supplementary file1 (DOCX 19 KB) [file 11604_2023_1458_MOESM1_ESM.docx]

**Supplementary Materials**

1. **Supplementary methods**
2. **Supplementary table**

**I．Supplementary methods**

**The specific definition and description of the features were as follows:**

(1) First-order histogram features. These features describe the central tendency, variability, uniformity, asymmetry, skewness and magnitude of the attenuation values in a given region of interest (ROI), disregarding the spatial relationship of the individual voxels.

(2) Morphologic features. Morphologic features describe the size and shape of a given ROI, without taking into account the attenuation values of its voxels.

(3) Gray Level co-existence Matrix (GLCM) features. A GLCM describes the number of times a voxel of a given attenuation value i is located next to a voxel of j.

(4) Gray-level size zone matrix (GLSZM) features. A GLSZM features describes gray level zones in a ROI, which are defined as the number of connected voxels that share the same gray level intensity.

(5) Gray Level range-matrix (GLRM) features. A GLRM features describes gray level runs, which are defined as the length in number of pixels, of consecutive pixels that have the same gray level value.

(6) Neighbouring Gray Tone Difference Matrix (NGTDM) features. A NGTDM features quantifies the difference between a gray value and the average gray value of its neighbours within distance δ.

(7) Gray Level Dependence Matrix (GLDM) features. A GLDM features quantifies gray level dependencies in an image. A gray level dependency is defined as the number of connected voxels within distance δ that are dependent on the center voxel.

**Ⅱ. Supplementary Table S1**

**Table S1.** Scanning parameters of abdominal triphasic enhanced CT in different instruments.

| Parameter | Discovery CT 750 HD | Philips ICT 128 |
| --- | --- | --- |
| Tube voltage, kVp | 120 | 120 |
| Tube current, mAs | 350 | 251 |
| Collimator width, mm | 40 | 80 |
| Rotation time, s | 0.5 | 0.5 |
| Screw pitch | 0.984:1 | 1.150:1 |
| Reconstructed layer thickness, mm | 1.25 | 1.25 |
